# Coping Flexibility and Health-Related Quality of Life Among Older Adults: The Compensatory Effect of Co-rumination

**DOI:** 10.3389/fpsyg.2019.00059

**Published:** 2019-01-23

**Authors:** Aleksandra Kroemeke

**Affiliations:** Department of Psychology, SWPS University of Social Sciences and Humanities, Warsaw, Poland

**Keywords:** coping, health-related quality of life, heterogeneity, older adults, latent class growth analysis

## Abstract

**Background:** Coping flexibility, defined as a wide range of coping strategies, may be a promising construct in determining coping effectiveness, especially in conjunction with a person-centered approach. However, no studies have focused on these issues. The study aimed to identify the distinct, multidimensional patterns of strategies for coping with chronic health conditions and their association with changes in physical and psychological health-related quality of life (HRQoL) among older adults over a one month period.

**Methods:** Coping strategies (brooding, reflection, co-rumination, and positive reappraisal) and HRQoL psychological and physical domains were assessed twice (at the baseline and one month later) among 210 older adults (age 76.12 ± 9.09 years, 66% women).

**Findings:** The parallel process analysis demonstrated the sample heterogeneity regarding coping. In multidimensional latent class growth analysis (MLCGA), four coping classes of overall strategies were identified: consistently low (46%), medium and decreasing (18%), medium and increasing (20%), and consistently high (16%). The last two can be considered the coping flexibility. Participants in the medium and increasing subgroup reported enhancement in HRQoL psychological domain, whereas members of the consistently high subgroup indicated its decrease. The favorable effects were related to an increase in co-rumination.

**Discussion:** The findings shed light on the longitudinal patterns of coping in older adults, showing that coping flexibility is more adaptive when it relies on modifying coping efforts rather than coping complexity. Co-rumination played a key role, compensated by the effect of maladaptive strategies.

## Introduction

Multimorbidity, defined as the co-occurrence of multiple diseases in the same individual, is progressively more common with age (Marengoni et al., 2011; Kirchberger et al., 2012; Salive, 2013). According to systematic review data, it ranges from 55 to 98% in elderly people and affects seniors’ well-being and quality of life (Marengoni et al., 2011; Salive, 2013). Despite the worldwide aging phenomenon and the commonness of chronic health problems with age, data regarding coping with health conditions among the elderly is still missing.

Chronic health problems in the elderly are little controllable (World Health Organization, 2011), hence, cognitive emotion-focused coping modes (e.g., rumination and positive reappraisal) seem to be important here (Lazarus and Folkman, 1984). Positive reappraisal is defined as the “cognitive strategy for reframing a situation to see it in a positive light” (Folkman, 1997, Folkman, 1212). Earlier studies suggest that positive reappraisal is associated with higher well-being and physical health (Schanowitz and Nicassio, 2006; Windsor, 2009; Masumoto et al., 2016), better social functioning (Schanowitz and Nicassio, 2006), and lower depressive symptoms or negative affect in older adults (Kraaij et al., 2002; Garnefski and Kraaij, 2006; Masumoto et al., 2016). Adaptive function of positive reappraisal is also identified in a recent meta-analysis (Nowlan et al., 2015).

On the contrary, rumination, defined as negative recurrent thoughts and/or images (Thomsen et al., 2005), is associated with a decrease in the quality of life for older adults and a worsening of their psychological, physical, and cognitive health (Thomsen et al., 2004; Segerstrom et al., 2010; Gan et al., 2015). Among ruminations, brooding and reflection stand out (Treynor et al., 2003). The first concerns passive, repetitive thinking about the situation and its comparison to currently inaccessible standards. Reflection, or so-called active rumination, is oriented to drawing constructive conclusions for the future. Studies demonstrate both constructs to have good construct validity (Treynor et al., 2003; Schoofs et al., 2010; Whitmer and Gotlib, 2011), also in elderly individuals (Rewston et al., 2007; Sütterlin et al., 2012). In seniors, brooding is related to lower life satisfaction and higher depression, while reflection is not related to adjustment (Sütterlin et al., 2012). Similar relations are observed as cross-sectional and longitudinal in younger samples (Burwell and Shirk, 2007; Marroquín et al., 2010; Verstraeten et al., 2010).

Rose (2002) introduced another dimension of rumination—co-rumination—which includes discussion of one’s own problems and negative emotions with other people. To the best of our knowledge, co-rumination in elderly people has not been investigated. Studies in young adults and adolescents demonstrated, on the one hand, that it is related to brooding and has similar, maladaptive effects. On the other hand, it may enhance intimacy between co-ruminating persons, thus bringing benefits (Rose et al., 2007).

Taken together, brooding is consistently linked to a poorer psychological outcome, and positive reappraisal has beneficial effects, while the effects of reflection and co-rumination remain unclear. According to Lazarus and Folkman’s (1984) stress and coping theory, one possible contributor to these mixed findings is that no single coping strategy is adaptive or maladaptive across all stressful conditions. Moreover, in particular stressful situations, no single strategy is taken exclusively, rather, a wide range of coping strategies is employed. Focusing on only one strategy may give misleading conclusions. For example, Aldao and Nolen-Hoeksema (2012) show that so-called adaptive strategies (e.g., positive appraisal or acceptance) have a weaker association with psychopathology than maladaptive ones (e.g., brooding or denial). Their study supports a compensatory hypothesis according to which adaptive strategies are especially effective when both adaptive and maladaptive strategies are used. This hypothesis is consistent with the idea of coping flexibility. According to the definition, “coping flexibility refers to intra-individual variability in the deployment of diverse coping strategies” promoting adjustment to stressful circumstance (Cheng et al., 2014, 3). The conceptualization of coping flexibility is not unequivocal, and there are several types of understanding this concept in the literature (for review: Bonanno and Burton, 2013; Cheng et al., 2014). Among the conceptualizations, those referring to the size of coping deployed (i.e., broad repertoire or a wide range of coping strategies; balanced profile or a moderate use of all coping strategies), matching the situation (i.e., cross-situation variability; strategy-situation fit), and self-evaluation of coping ability and the ability to monitor and correct coping behaviors are mentioned (Bonanno and Burton, 2013; Cheng et al., 2014). One of the simplest but still little explored conceptualizations of coping flexibility is a broad repertoire or number of strategies deployed in stressful situations (Bonanno and Burton, 2013; Cheng et al., 2014). Previous cross-sectional studies indicated that a broad repertoire of coping contributes to lower psychological distress (Lam and McBride-Chang, 2007), depression, and anxiety (Lougheed and Hollenstein, 2012) among adolescents. Using a clustering approach, a flexible coping group showed lower depressive symptoms and self-assessed their coping as more effective cross-sectionally and during follow-up (Cheng, 2001). Using a different conceptualization that is a well-balanced coping profile approach to flexible coping characterized by a moderate use of all coping strategies, coping flexibility was related to less grief and more posttraumatic growth (Cohen and Katz, 2015). In another study, adults with complicated grief show less coping flexibility (Burton et al., 2012). Longitudinally, a well-balanced coping profile predicts positive adaptation to potentially traumatic events (Galatzer-Levy et al., 2012).

To the best of our knowledge, coping flexibility defined as coping repertoire in elderly people has not been investigated, although stronger effects of coping flexibility are observed in older individuals, mainly using balanced profile conceptualization (Cheng et al., 2014). Moreover, previous studies rarely have a longitudinal design, which does not allow for testing time-variability and temporal or dynamic relationships between different coping strategies, including coping flexibility, and adjustment trajectories (Bonanno and Burton, 2013). Another issue is the nature of coping flexibility. It is mostly treated as trait-like and consequently measured using coping style questionnaires (Bonanno and Burton, 2013). However, coping is an idiosyncratic process, varying with the person, situation, and time (Lazarus and Folkman, 1984). Thus, measurement of a flexible coping mode should instead be based on non-trait-like manners (i.e., using coping strategies in the long run) and a proper methodology accounting for the coping multidimensional heterogeneity. Also, previous studies omitted the variability of study participants treating the sample as homogeneous and based on the average results of coping for an entire group, according to the so-called variable-centered approach (Laursen and Hoff, 2006). In such studies, the operationalization of broad repertoire refers to a sum score for all measured coping modes (a higher score indicates more flexible coping). Meanwhile, a more promising approach to studying coping flexibility as a broad repertoire may be a person-centered approach allowing for identification of subgroups of people characterized by a high internal similarity of coping patterns (low or high scores) but significant differences between subgroups (Laursen and Hoff, 2006).

This study attempted to address the above issues, aiming to identify the distinct multidimensional intra-individual patterns of cognitive emotion-focused coping strategies with chronic health conditions among older adults over one month and to evaluate whether different coping trajectory subgroups could determine one-month changes in seniors’ physical and psychological health-related quality of life (HRQoL). Based on Lazarus and Folkman’s (1984) stress and coping theory, HRQoL was considered a coping outcome. A one-month time frame was chosen as sufficient to observe significant health and psychological functioning changes, especially in the elderly (World Health Organization, 2011). In addition to investigating the advantages of a broad repertoire of cognitive emotion-focused coping strategies in older adults, this study adds to the literature by employing a longitudinal state-like and person-centered approach to coping flexibility. The study hypothesizes that four different patterns of coping, including brooding, reflection, co-rumination, and positive reappraisal, would be found: stable (low vs. high, flexible groups) and change (decreasing vs. increasing groups; Hypothesis 1), and that subgroup membership would predict HRQoL changes. In particular, subgroups with flexible coping and with increased coping modes would strengthen HRQoL over time (Hypothesis 2).

## Materials and Methods

### Participants and Procedure

The participants were 210 elderly people (66% women) aged 60–100 years (*M* = 76.12 ± 9.09) who were assessed twice: at the baseline (T1; *N* = 277) and one month later (T2; *N* = 210). Of the participants, 65% (*T*_1_ = 180; *T*_2_ = 137) were residents of nursing homes (range of stay from one to 303 months, *M* = 61.60 ± 60.16 months), and 35% (*T*_1_ = 97; *T*_2_ = 73) attended daily seniors’ clubs (for about 5–6 h each day). Inclusion criteria were as follows: ages ≥ 60, lack of cognitive disorders (no diagnosis of dementia or mild cognitive impairments, and efficient cognitive functioning confirmed by the care facility personnel), and a lack of serious acute somatic illness that may significantly affect results. The final (T2) sample characteristic is shown in Table 1. Most participants were single (unmarried, widowed, or divorced), had achieved secondary or primary education, and had not used medical care for the previous six months. Participants suffered from more than four coexisting chronic illnesses and took, on average, six medicines per day. The average functional status of participants was relatively high, based on the Katz Index of Activities of Daily Living (ADL) (Katz et al., 1963) and the Lawton Instrumental Activities of Daily Living (IADL) Scale (Lawton and Brody, 1969).

**Table 1:** Sample characteristics (*N* = 210).

Demographic characteristics at baseline	*n*	(%)
Male	72	(34%)
Cohabiting	40	(19%)
Subjective economic status		
Above average	54	(26%)
Average	132	(63%)
below average	24	(11%)
Residents of nursing homes	137	65%

	***M***	**(*SD*), range**

Age (years)	76.12	(9.09), 60–100
Education (years)	10.92	(3.45), 2–18

**Medical characteristics at baseline**	***n***	**(%)**

Medical comorbidities		
None	5	(2%)
1	20	(10%)
2	27	(13%)
3	28	(13%)
4 or more	130	(62%)
Medical care for the previous 6 months	82	(39%)

	***M***	**(*SD*), range**

Medical comorbidities	4.51	(2.64), 0–12
Daily number of medication use	6.44	(4.47), 0–22
ADL (Cronbach’s α = 0.71)	5.63	(0.90), 0–6
IADL (Cronbach’s α = 0.85)	20.97	(3.88), 9–24

*ADL, Katz Index of Activities of Daily Living; *IADL*, Lawton Instrumental Activities of Daily Living Scale*.

The institution did not differentiate participants by controlled variables, except for age, education, and functional status. The residents of nursing homes (*M*_NH_ = 77.83 ± 8.98) were significantly older than those attending the daily seniors’ clubs (*M*_SC_ = 72.96 ± 8.47, *t*_209_ = -3.54, *p* < 0.001), less educated (*M*_NH_ = 10.53 ± 3.49 vs. *M*_SC_ = 11.64 ± 3.27, *t*_209_ = 2.24; *p* = 0.026), and rated their functional status worse (ADL: *M*_NH_ = 5.48 ± 1.07 vs. *M*_SC_ = 5.89 ± 0.31, *t*_174.17_ = 3.22, *p* = 0.002; IADL: *M*_NH_ = 19.98 ± 4.10 vs. *M*_SC_ = 22.80 ± 2.59, *t*_203.84_ = 5.36; *p* < 0.001).

The study was conducted in accordance with the recommendations of the SWPS University of Social Sciences and Humanities Ethics Committee, and the University Ethics Committee approved the protocol (decision no. 22/2012). All participants gave written informed consent in accordance with the Declaration of Helsinki. Participation in the study was voluntary. At both measurement points, participants completed questionnaires to evaluate coping strategies, quality of life, and health indicators. Taking into consideration the specificity of the study group, abbreviated or experimental versions of the research tools were used (i.e., consisting of two to seven items).

The sample attrition analyses indicated that the missing data was similar in the nursing home and senior club groups (approximately 23%); respondents (*n* = 212) and non-respondents (*n* = 65) did not differ in sociodemographic and medical variables, or main study variables except age (non-respondents were older than respondents; χ^2^_1_ = 15.92, *p* < 0.001, OR = 0.94).

### Measures

#### Coping Strategies

Four kinds of coping strategies with health problems were analyzed twice: brooding, reflection, co-rumination, and positive reappraisal. Brooding (e.g., “*I think ‘Why do I have health problems other people don’t have’*”) and reflection (e.g., “*I think about why I feel this way*”) were measured using the Polish version of four Ruminative Response Styles items (RRS) (Treynor et al., 2003; Whitmer and Gotlib, 2011) specially modified for this study (the instruction and items referred to the somatic and health problems in the last week). Co-rumination (e.g., “*When I talk with someone I tell him/her every detail about my health problems*”) was measured using two modified (in the manner as above) Co-Rumination Questionnaire (CRQ) items (Rose, 2002; Davidson et al., 2014). Positive reappraisal (e.g., “*I’ve been trying to see it in a different light, to make it seem more positive.*”) was measured using the Polish version of two items from the abbreviated situational version of COPE (Brief COPE) (Carver, 1997). All statements were assessed on a 4-point scale from 1 (*I haven’t been doing this at all*) to 4 (*I’ve been doing this a lot*). The higher the results, the greater the intensity of a given strategy. All coping indicators had acceptable reliability coefficients (see Table 2), considering the double-item nature.

**Table 2:** Descriptive statistics and bivariate correlations (*N* = 210).

	*M (SD)*	Timecomparison, *t*	Cronbach’s α	2.	3.	4.	5.	6.	7.	8.	9.	10.	11.	12.
(1) Brooding T1	4.40	1.67	0.67	0.58**	0.72**	0.48**	0.43**	0.38**	0.30**	0.17*	-0.27**	-0.22**	-0.31**	-0.29**
	(2.17)	
(2) Brooding T2	4.18		0.68		0.50**	0.71**	0.19**	0.41**	0.16*	0.22**	-0.26**	-0.33**	-0.32**	-0.35**
	(2.17)	
(3) Reflection T1	4.89	1.39	0.69			0.50**	0.32**	0.33**	0.21**	0.15*	-0.25**	-0.22**	-0.25**	-0.25**
	(2.15)	
(4) Reflection T2	4.68		0.76				0.17*	0.42**	0.14*	0.26**	-0.35**	-0.33**	-0.32**	-0.30**
	(2.27)	
(5) Co-rumination T1	4.29	1.09	0.88					0.54**	0.29**	0.19**	-0.03	-0.07	-0.04	-0.15*
	(2.34)	
(6) Co-rumination T2	4.13		0.89						0.21**	0.29**	-0.16*	-0.19**	-0.07	-0.14*
	(2.21)	
(7) Positive reappraisal T1	5.42	3.74***	0.66							0.57**	0.09	0.06	0.19**	0.19**
	(1.96)	
(8) Positive reappraisal T2	4.92		0.81								0.05	-0.01	0.19**	0.13
	(2.15)	
(9) Physical HRQoL T1	22.97	2.81**	0.73									0.66**	0.57**	0.48**
	(5.07)	
(10) Physical HRQoL T2	22.14		0.77										0.53**	0.63**
	(5.26)	
(11) Psychological HRQoL T1	20.89	3.11**	0.73											0.75**
	(4.27)	
(12) Psychological HRQoL T2	20.21		0.75											
	(4.27)	

*^∗^*p* < 0.05; ^∗∗^*p* < 0.01; ^∗∗∗^*p* < 0.001*.

#### Health-Related Quality of Life Physical and Psychological Domain

The physical (seven items, e.g., “*How much do you need medical treatment to function in your daily life?*”) and psychological domain (six items, e.g., “*How much do you enjoy life?*”) of HRQoL was measured twice using the Polish version of the short form of the World Health Organization Quality of Life Questionnaire (WHOQOL-BRIEF) (Skevington et al., 2004). All questions were assessed on a 5-point scale from 1 (*Not at all*) to 5 (*An* e*xtreme amount/Extremely*). Raw domain scores for the WHOQOL-BRIEF were used. The higher the results, the greater the HRQoL of a given domain. Both indicators had acceptable reliability coefficients and were comparable to validation studies (Skevington et al., 2004; Jaracz et al., 2006).

### Statistical Analysis

Descriptive statistics were computed using IBM SPSS (IBM Corp.; Armonk, NY, United States) version 24. The dataset contains no missing values (see Supplementary Data S1). To identify heterogeneous classes of the overall coping strategies among elderly people, multidimensional latent class growth analysis, (MLCGA) (Wickrama et al., 2016), was conducted using Mplus statistical package version 8 (Muthén and Muthén, 1998). The minimum recommended sample size for this analysis is 5 × 2^k^, where *k* is the number of the variables in the analysis (Dolnicar, 2002). The minimum acceptable sample size was determined to be *N* = 80 (5 × 2^4^). First, the parallel process latent growth curve model (PPM) was estimated to examine the trajectory of all coping strategies simultaneously. Next, the unconditional MLCGA was applied. MLCGA is essentially a special case of multidimensional growth mixture modeling (MGMM) in which the growth parameters are assumed to be invariant within classes (Muthén and Muthén, 2000; Wickrama et al., 2016). The maximum likelihood with robust standard errors was used as an estimator (Muthén and Muthén, 1998). Factor loadings corresponded directly to the time interval (T1 was set to 0 and T2 to 1). The linear slope was estimated. Models fitting between one and six classes were run successively. The determination of the appropriate classification was based on the Bayesian Information Criterion (BIC), the Sample-Size Adjusted Bayesian Information Criterion (SSABIC), the Bootstrap Likelihood Ratio Test (BLRT), the adjusted Lo-Mendell-Rubin Likelihood Ratio Test (LMR-LRT), entropy value, and practical usefulness of the latent trajectory classes (Wickrama et al., 2016). The model with the lower BIC and SSABIC values, greater entropy value (closer to 1), and significant BLRT and LMR-LRT tests (*p*s < 0.05 indicated that the estimated model is preferable over a model with one fewer class) indicated good fitting (Wickrama et al., 2016). After identifying the multidimensional coping classification, the conditional MLCGA was estimated. Distal continuous outcomes, i.e., changes in physical and psychological HRQoL (subtracting T1 from T2 scores), were examined using Lanza et al.’s approach (Lanza et al., 2013). This model-based approach does not influence MLCGA classification.

## Results

Descriptive statistics and correlations between the variables are presented in Table 2: The level of both HRQoL domains significantly decreased in time. Generally, coping strategies were significantly related to HRQoL domains, except for co-rumination. The effect sizes of these results were low to medium.

Results of PPM indicated stability of coping strategies in the whole sample on average (brooding: intercept = 4.39, *SE* = 0.15, *p* < 0.001; slope = -0.22, *SE* = 0.14, *p* = 0.102; reflection: intercept = 4.88, *SE* = 0.15, *p* < 0.001; slope = -0.21, *SE* = 0.15, *p* = 0.171; co-rumination: intercept = 4.28, *SE* = 0.16, *p* < 0.001; slope = -0.16, *SE* = 0.15, *p* = 0.286), except positive reappraisal, which decreased over time (intercept = 5.41, *SE* = 0.14, *p* < 0.001; slope = -0.49, *SE* = 0.13, *p* < 0.001). For all coping strategies, the absolute values of both skewness and kurtosis were below 1, which allows for an assumption of normal distribution (Gravetter and Wallnau, 2013). Poor model fit indicators indicated sample heterogeneity (-2LL = -3389.32, BIC = 6949.74, SSABIC = 68489.34, χ^2^ = 88.26, *p* < 0.001, χ^2^/*df* = 7.36, RMSEA = 0.174, CFI = 0.85, TLI = 0.66, SRMR = 0.18).

### Unconditional MLCGA

Table 3 shows the fit indicators for coping strategies in multidimensional LCGA. The adjusted LMR-LRT test supported the 2-class classification; however, the test for the model with three and four classes was marginally significant. The BIC value indicated the 4-class model. The SSABIC value, entropy measure, and BLRT test supported the 6-class model, however, in this classification only a few participants represented two classes. Taking this into account, the 4-class solution was chosen as a more parsimonious solution, given that it was characterized by a satisfactory BIC value, marginally significant LMR-LRT test, and sufficient class sizes.

**Table 3:** Results of unconditional MLCGA—fit indexes (*N* = 210).

Fit Statistics	2 Classes	3 Classes	4 Classes	5 Classes	6 Classes
-2LL (No. of parameters)	-3376.56 (37)	-3340.15 (46)	-3288.66 (55)	-3267.19 (64)	-3253.25 (73)
BIC	6950.97	6926.27	**6871.42**	6876.59	6896.83
SSABIC	6833.73	6780.51	6697.14	6673.81	**6665.53**
Entropy	0.948	0.897	0.927	0.940	**0.956**
Adj. LMR-LRT (*p*)	**171.47^∗∗∗^**	71.34^∧^	100.88^∧^	42.07	27.32
BLRT (*p*)	-3464.08^∗∗∗^	-3376.56^∗∗∗^	-3340.15^∗∗∗^	-3288.66^∗∗∗^	**-3267.19^∗∗∗^**
Class size (%)
C1	69 (33%)	96 (46%)	97 (46%)	43 (20%)	92 (44%)
C2	141 (67%)	72 (34%)	37 (18%)	31 (15%)	5 (2%)
C3		42 (20%)	34 (16%)	38 (18%)	3 (1%)
C4			42 (20%)	94 (45%)	37 (18%)
C5				4 (2%)	40 (19%)
C6					33 (16%)

*Best fit indicates are in bold. ˆ*p* = 0.05; ^∗^*p* < 0.05; ^∗∗^*p* < 0.01; ^∗∗∗^*p* < 0.001*.

**Figure 1:**
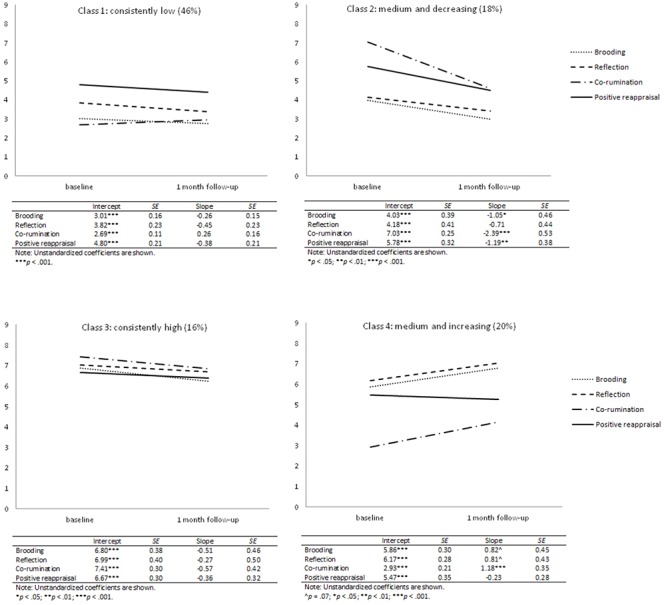
Mean coping patterns for multidimensional latent classes. Plots refer to sample means, while tables refer to estimated means.

In 4-class classification, most of the sample (*n* = 97, 46%) belonged to the subgroup with low and stable all coping strategies (Figure 1). The second subgroup (*n* = 37, 18%) included seniors with medium and decrease in time coping strategies, particularly brooding, co-rumination, and positive reappraisal. In the third subgroup (*n* = 34, 16%), participants showed relatively high and stable all coping strategies. The last class (*n* = 42, 20%) comprised participants with medium and increase in time coping scores, particularly concerning co-rumination.

### Conditional MLCGA

Table 4 shows the results of testing changes in the HRQoL domains as distal outcomes of coping classification. The conditional model—with continuous distal outcomes—significantly improved model fit as compared to the unconditional model; -2LL = -3158.59, BIC = 6609.13, SSABIC = 6434.88. Only changes in the psychological HRQoL domain distinguished coping classes. Participants in the medium and increasing coping subgroup reported improvement in psychological HRQoL over time compared to participants from the consistently low, the medium and decreasing, and the consistently high coping subgroups. The consistently high coping subgroup reported a decrease in psychological HRQoL compared to the consistently low subgroup.

**Table 4:** Results of the conditional MLCGA—the mean structure of continuous distal outcomes: changes in physical and psychological (T2-T1) HRQoL domain.

Outcomes	Class1: consistently low	Class 2: medium and decreasing	Class 3: consistently high	Class 4: medium and increasing	Overall Chi-square test
ΔPhysical HRQoL	-0.92	-0.04	-1.96	-0.67	3.85
ΔPsychological HRQoL	-0.49	-0.82	-2.18	0.64	15.93^**a,b,c,d^

*Unstandardized coefficients are shown. The 4-class MLCGA was used. ^∗^*p* < 0.05; ^∗∗^*p* < 0.01; ^∗∗∗^*p* < 0.001. ^*a*^class 1 vs. class 3, *p* < 0.05. ^*b*^class 1 vs. class 4, *p* < 0.05. ^*c*^class 2 vs. class 4, *p* < 0.05. ^*d*^class 3 vs. class 4, *p* < 0.001*.

## Discussion

In this study, the heterogeneity of longitudinal patterns of cognitive emotion-focused coping strategies and their effectiveness in psychological and physical HRQoL over one month were examined among older adults. This approach was applied to investigate the beneficial effects of the coping flexibility hypothesis in emotion-focused strategies. The findings revealed heterogeneity in four coping patterns and their association with HRQoL psychological domain changes.

In line with predictions (Hypothesis 1), four distinct coping trajectories were found. Almost half of the seniors (46%) reported a stable low-level of brooding, reflection, co-rumination, and positive reappraisal over one month (consistently low subgroup). Participants from the medium and increasing subgroup (20%) demonstrated all coping strategies at the medium level at baseline and then increases one month later. More specifically, only the significant increase in co-rumination was noted. Another subgroup (18%) showed medium all coping strategies with the decrease in time, namely regarding brooding, co-rumination, and positive reappraisal (medium and decreasing subgroup). The smallest group (16%) reported relatively high consistently stable coping with health conditions in time (consistently high subgroup). Two of these subgroups can be considered as fitting the coping flexibility concept, namely, the consistently high subgroup and medium and increasing subgroup.

The largest group was seniors with low all types of coping, which would indicate that the respondents undertook cognitive emotion-focused coping in a small range, at least in relation to the studied strategies. Subgroups with coping flexibility were in the minority, constituting just 36% of the sample. These findings, regarding the coping repertoire, were not previously reported in the literature. Classification by Cheng (2001) revealed similar results, although this classification was based on the goodness-of-fit definition of coping flexibility. It was concerned with the interaction between perceived event controllability (controllably vs. uncontrollably) and coping used (problem- vs. emotion-focused). In three studies, flexible subgroups comprised 19 to 30% of the samples, while the largest subgroup (38–67%) preferred to use problem-focused coping independent from the controllability assessment (inflexible or inconsistent groups). It cannot be excluded that the respondents in the current study also preferred problem-focused strategies, despite objectively small controllability of their health conditions (World Health Organization, 2011), or they used another emotion-focused coping, such as acceptance or humor, or other types of coping, like avoidance. For comparison, problem-focused and meaning-focused coping, as well as controlling existing diseases, were mainly reported by older individuals in a quantitative study (Löffler et al., 2012). Another explanation for the current study result is connected with age-related changes in perceived stress and coping, namely that coping efforts decrease with age even if stressful conditions are perceived (Felton and Revenson, 1987; Aldwin et al., 1996).

The consequences of coping subgroup membership are particularly interesting. Contrary to expectation (Hypothesis 2), coping flexibility was not distinctly beneficial. Somewhat surprisingly, results showed a consistently high subgroup decrease in psychological HRQoL over one month compared to the consistently low group. The cause may be that the permanently high coping efforts and the broad repertoire involve psychological costs. Treating coping as a resources management process (Hobfoll, 2002), coping costs concern resource loss or depletion after investing and are more probable if the resources expended in the coping process exceed its outcomes. The detrimental effect of coping flexibility in the current study may also be related to particular strategies that are a part of a combination. For example, with brooding (which has an unequivocal, negative effect; Burwell and Shirk, 2007; Marroquín et al., 2010; Verstraeten et al., 2010; Sütterlin et al., 2012) or with reflection (conceptually a more adaptive mechanism; Treynor et al., 2003), which was less effective in this study as related to lower HRQoL in the whole sample.

Another possible explanation of the reduction of HRQoL psychological domain in the consistently high subgroup may be no equivalence of particular coping strategies, to which Aldao and Nolen-Hoeksema (2012) also referred. Rather than depending on using a specific strategy, the overall effect of coping depends on the use of both adaptive and maladaptive strategies simultaneously. For the overall gain, maladaptive coping efforts (e.g., brooding or reflection) would have to be compensated by adaptive coping modes (e.g., positive reappraisal or co-rumination). The compensation hypothesis is supported by the beneficial effect of belonging to the medium and increasing subgroup. Participants following this coping pattern reported psychological HRQoL enhancement. Indeed, only co-rumination improved in this subgroup indicating the adaptability of this coping mode and the ability to mitigate the negative effect of maladaptive strategies. To date, results of co-rumination appear to be mixed (Rose et al., 2007). A novel finding in this study is the broadening of knowledge about coping flexibility. “More” does not always mean “better.” Possibly, either key strategies are required in coping repertoire, or a distinct combination of various behaviors is needed to cope effectively. Of note, the intercept of co-rumination in the medium and increasing subgroup was on a similar level to the consistently low group. The adaptability of the former coping path may result in a correction of coping: take effort on the social disclosure, thus building the positive affect. It refers to the responsiveness to feedback types of coping flexibility consisting of monitoring and modifying coping behavior as needed (Bonanno and Burton, 2013). This result can also be considered as confirmation of the beneficial effect of social exchange in old age (Newsom et al., 2003; Kroemeke and Gruszczynska, 2016).

Surprisingly, positive reappraisal was not a crucial strategy, although its adaptive function was repeatedly reported (Kraaij et al., 2002; Garnefski and Kraaij, 2006; Schanowitz and Nicassio, 2006; Windsor, 2009; Nowlan et al., 2015; Masumoto et al., 2016). Additionally, physical HRQoL was not predicted. Cognitive emotion-focused strategies, according to their function (Lazarus and Folkman, 1984), modified only the individual affect, not subjective health condition assessment. Despite this, HRQoL decreased over one month in the whole sample, indicating the worsening of both somatic and psychological senior conditions.

A practical implication of note in these findings can be found in the building and multiplication of coping resources for seniors to prevent their loss or depletion, especially in groups exposed to permanent health-related stress and coping. Another practical implication worth noting is the need to arouse adaptive coping modes. These could mitigate the effect of maladaptive strategies all at once, while flexible coping relies on modifying coping efforts rather than tackling its complexity. Finally, older people may especially benefit from sociable strategies; thus creating opportunities for social interactions would be desirable.

The findings have important theoretical and practical implications; however, several limitations should be noted. First, the sample has to be considered as non-homogeneous, as it included both seniors staying in nursing homes and those attending the senior clubs. However, this potential detractor was controlled statistically by testing the institution as a predictor of coping classification. No effect was observed as the institution turned out to be an insignificant predictor of coping classification (see Supplementary Table S1). Furthermore, including both seniors groups is a unique strength of this project as samples in studies of older adults most often include students of the University of the Third Age. Second, the study covered only a one month period. Although this period is justifiable to assess health and HRQoL changes, and associated with them coping (World Health Organization, 2011), a longitudinal study with a larger number of measurements is needed to bring more accurate data on coping trajectories and their outcomes in both seniors in institutional and natural surroundings. Third, despite the longitudinal design of the study, the conditional model results should not be treated regarding their cause-and-effect relationships. Rather, parallel processes of both coping and HRQoL changes were investigated. Based on the transactional stress and coping model (Lazarus and Folkman, 1984), the predictive effects of coping changes on coping outcomes changes (HRQoL) was investigated. The reverse direction of dependences between variables cannot be ruled out. Changes in coping efforts could be determined by changes in perceived quality of life, e.g., emotional state or physical burden. Additionally, various domains of HRQoL could have a different function in coping process with a health condition in older adults. For example, changes in physical symptoms (physical HRQoL) could determine changes in coping with health problems, which, in turn, could predict the emotional state (psychological HRQoL). This would explain the lack of effect on physical HRQoL in this study; hence, further studies are needed in this area. Fourth, specific coping strategies relating to the cognitive level were analyzed; thus, further studies on the effectiveness of coping flexibility in the elderly should investigate a broader spectrum of coping modes with health problems (e.g., behavioral level, and approach and avoidance strategies).

Despite the limitations, the current study is the first to use a latent class growth approach to examine the coping flexibility, defined as a wide range of coping strategies, and its outcomes in the older adult sample. The findings highlight the heterogeneity of patterns of coping with health conditions with ambiguous effects of coping flexibility, indicating the practical implications of these results.

## Author Contributions

AK contributed to the conception and design of the work and contributed to the acquisition, analysis, and interpretation of data for the work. AK drafted the work and revised it critically for important intellectual content. AK approved the final version to be published and also agreed to be accountable for all aspects of the work in ensuring that questions related to the accuracy or integrity of any part of the work are appropriately investigated and resolved.

## Conflict of Interest Statement

The author declares that the research was conducted in the absence of any commercial or financial relationships that could be construed as a potential conflict of interest.

## Supplementary Material

DATA S1 Coping in old age-coping flexibility. Dataset for “Coping flexibility and health-related quality of life among older adults: compensatory effect of co-rumination” in.sav format.

TABLE S1 The coefficients of predictors of the 4-class coping classification from the auxiliary 3-step approach (*N* = 210).
