# Supplementary material for: Coping Flexibility and Health-Related Quality of Life Among Older Adults: The Compensatory Effect of Co-rumination
Source: Front Psychol. 2019 Jan 23;10:59. doi: 10.3389/fpsyg.2019.00059 (PMC6351461; doi:10.3389/fpsyg.2019.00059)
Supplement: TABLE S1 — The coefficients of predictors of the 4-class coping classification from the auxiliary 3-step approach (N = 210). [file Table_1.docx]

***Supplementary Material***

**Coping flexibility and health-related quality of life among older adults: compensatory effect of co-rumination**

**Aleksandra Kroemeke**

**Correspondence:**

Aleksandra Kroemeke

[akroemeke@swps.edu.pl](mailto:akroemeke@swps.edu.pl)

Table S1. The coefficients of predictors of the 4-class coping classification from the auxiliary 3-step approach (*N*=210).

| **Predictors** | **Estimate** | ***SE*** | ***p*** |
| --- | --- | --- | --- |
| Parameterization using reference class 4 | | | |
| **Class 1** | | | |
| Age | .05 | .03 | .049 |
| Gender | -.31 | .44 | .485 |
| Education | .06 | .06 | .333 |
| Institution | .89 | .50 | .072 |
| ADL T1 | .13 | .29 | .659 |
| IADL T1 | .02 | .08 | .773 |
| **Class 2** | | | |
| Age | .02 | .03 | .567 |
| Gender | -.73 | .53 | .167 |
| Education | -.19 | .09 | .029 |
| Institution | .88 | .56 | .113 |
| ADL T1 | -.16 | .28 | .571 |
| IADL T1 | .05 | .08 | .513 |
| **Class 3** | | | |
| Age | .06 | .03 | .066 |
| Gender | -.24 | .59 | .683 |
| Education | -.10 | .08 | .208 |
| Institution | .41 | .64 | .525 |
| ADL T1 | .37 | .25 | .148 |
| IADL T1 | -.11 | .07 | .125 |
| Parameterization using reference class 3 | | | |
| **Class 1** | | | |
| Age | -.00 | .02 | .902 |
| Gender | -.07 | .53 | .894 |
| Education | .16 | .07 | .028 |
| Institution | .48 | .56 | .384 |
| ADL T1 | -.24 | .27 | .384 |
| IADL T1 | .13 | .07 | .072 |
| **Class 2** |  |  |  |
| Age | -.04 | .03 | .230 |
| Gender | -.49 | .62 | .429 |
| Education | -.08 | .10 | .394 |
| Institution | .47 | .64 | .461 |
| ADL T1 | -.52 | .27 | .050 |
| IADL T1 | .16 | .07 | .028 |
| Parameterization using reference class 2 | | | |
| **Class 1** | | | |
| Age | .03 | .027 | .212 |
| Gender | .42 | .462 | .360 |
| Education | .24 | .079 | .002 |
| Institution | .01 | .474 | .976 |
| ADL T1 | .29 | .289 | .320 |
| IADL T1 | -.03 | .079 | .711 |

Note: Unstandardized coefficients are shown. The 4-class MLCGA was used.

*ADL*, Katz Index of Activities of Daily Living; *IADL*, Lawton Instrumental Activities of Daily Living Scale.
